# Enhanced insulin activity achieved in VDRa/b ablation zebrafish

**DOI:** 10.3389/fendo.2023.1054665

**Published:** 2023-02-13

**Authors:** Ruolan Liu, Yao Lu, Xuyan Peng, Jingyi Jia, Yonglin Ruan, Shengchi Shi, Tingting Shu, Tianhui Li, Xia Jin, Gang Zhai, Jiangyan He, Qiyong Lou, Zhan Yin

**Affiliations:** ^1^ State Key Laboratory of Freshwater Ecology and Biotechnology, Institute of Hydrobiology, Chinese Academy of Sciences, Wuhan, China; ^2^ College of Advanced Agricultural Sciences, University of Chinese Academy of Sciences, Beijing, China; ^3^ The Laboratory of Ophthalmology and Vision Science, Department of Ophthalmology, The First Affiliated Hospital of Zhengzhou University, Zheng Zhou, China; ^4^ The Innovative Academy of Seed Design, Chinese Academy of Sciences, Beijing, China

**Keywords:** 1α,25(OH)2VD3, VDRs, insulin, lipolysis, glucose metabolism

## Abstract

**Introduction:**

1α,25-dihydroxyvitamin D3 (1α,25[OH]_2_VD_3_) is a hormone known for its key roles in calcium absorption and nutrient metabolism. In teleost fishes, 1α,25(OH)_2_VD_3_ insufficiency causes impaired glucose metabolism and lipid oxidation. However, the cascade and mechanisms of 1α,25(OH)_2_VD_3_ and the vitamin d receptor (VDR) signaling are unclear.

**Results:**

In this study, two genes (*vdra* and *vdrb*) encoding paralogs of VDRs were genetically knocked out in zebrafish. Growth retardation and accumulated visceral adipose tissue have been observed in *vdra* ^-/-^;*vdrb* ^-/-^ deficient line. In the liver elevated accumulation of triglycerides and suppressed lipid oxidation were detected. Morover significantly elevated 1α,25(OH)_2_VD_3_ levels were detected in *vdra^-/-^;vdrb^-/-^* zebrafish due to cyp24a1 transcription repression. Furthermore VDRs ablation Enhanced insulin signaling including elevated *insulin/insra* trancriptional levels, glycolysis, lipogenesis and promoted AKT/mTOR activity.

**Discussion:**

In conclusion, our present studies provides a zebrafish model with an elevated 1α,25(OH)_2_VD_3_ levels *in vivo*. The 1α,25(OH)_2_VD_3_/VDRs signaling promote lipid oxidation activity. However 1α,25(OH)_2_VD_3_ activity of regulation of glucose homeostasis through Insulin/Insr was independent of nuclear VDRs in teleosts.

## Introduction

Traditionally, 1α,25(OH)_2_VD_3_ is a well-known steroid hormone for its crucial functions regulating mineral ion homeostasis in mammals (1–3). In zebrafish, a model teleost fish, 1α,25(OH)_2_VD_3_ has been suggested to be involved in amino acid metabolism, inner ear and heart development, calcium handling, and cartilage development (4–10). In zebrafish mutants with deficient in *cyp2r1*, an enzyme involved in vitamin D_3_ activation, fatty acid oxidation in visceral adipose tissue (VAT) is promoted by 1α,25(OH)_2_VD_3_ in our laboratory (8). Recently, Shao et al. (2022) revealed the role of 1α,25(OH)_2_VD_3_ in the regulation of insulin and glucose metabolism with *cyp2r1*-deficient models (11). These studies indicated the diversity of physiological functions of vitamin D_3_ signaling developed over its long evolution.

The vitamin D3 and the metabolites developed functional versatility in energy metabolism, innate immune and skeletal development during the evolution. The action of 1α,25(OH)_2_VD3 has been suggested to be mediated by the nuclear vitamin D receptor (VDR), which form as a heterodimer with the retinoid X receptor, through specific sequences (VDR responsive elements [VREs]) on its target genes (1). A single *vdr* gene has been reported in most mammals, but two *vdr* paralogs, *vdra* and *vdrb*, have been identified in teleost fish; these paralogs arose after a whole-genome duplication event in teleosts (12). The functional diversity in the role of these two zebrafish VDRs in craniofacial cartilage development has been reported using a morpholino-based knockdown approach (10). Intriguingly, potent mitogenic effects on embryonic and adult cardiomyocytes have been demonstrated *in vivo* in *vdra* and *vdrb* double-knockout zebrafish, without effects on calcium handling, as stated recently (9). The metabolic functions of the vitamin D3 signaling was fascinating for the adipose tissue homeostasis and insulin action regulation. It was appealing to clarify the VDR functional diversity in the metabolic control using genome editing technology. Furthermore it is of interest to further clarify the effects of vitamin D_3_ signaling on mineral ion homeostasis and nutrient metabolism *in vivo* in zebrafish *vdr* mutants.

In the liver the enzymes CYP2R1 and CYP27A1 (not in human) convert vitamin D3 into 1,25(OH)_2_D3, which is the most stable vitamin D metabolite in serum. In the previous study, *cyp2r1*-defecient fish have moderate phenotypes in the skeletal system and craniofacial bones. The plasma levels of 1,25(OH)_2_D3 was significantly decreased however constitutive levels of 1,25(OH)_2_D3 still existed. To investigate the function of vitamin D3 signaling in skeletal system development two independent *vdra*- and *vdrb*-deficient zebrafish strains were generated using CRISPR/Cas9. Here we examined the multiple steps of vitamin D3 signaling to investigate the functional diversification of vitamin D3. The physiological phenotypes were analyzed and compared with the previous cyp2r1 ablation models. Intriguingly, the plasma levels of 1,25(OH)_2_D3 and the activity of Insulin signaling were promoted significantly in the VDRs ablation models. The objective of this study was to elucidate the underlying mechanisms of VD3/VDRs action.

## Materials and methods

### Zebrafish maintenance

Zebrafish (AB strains) were raised in a circulating aquarium system at 28.5°C with a 14-h light/10-h dark rhythm according to standard procedures. The animals were fed newly hatched brine shrimp (*Artemia salina*) three times at 8 am, 1 pm and 7 pm. Embryos were obtained through natural spawning and cultured at 28.5°C in egg water containing 0.006% ocean salt. The developmental stages of embryos were determined as previously described (13). Since the present study were performed using the adult zebrafish. To avoid ovulation cycle bias on lipid and glucose metabolism, the male zebrafish was used only in the study. All fish experiments were conducted in accordance with the Guiding Principles for the Care and Use of Laboratory Animals and were approved by the Institute of Hydrobiology, Chinese Academy of Sciences (Approval ID: IHB2013724).

### Establishment of *vdra* and *vdrb* knockout lines

The *vdra* and *vdrb* knockout lines were generated using CRISPR/Cas9 technology (14). Guide RNAs were designed using an online software tool (15) and were transcribed from PCR productsPCR products introduced a T7 promoter and gDNA sequence in the primer using the pMD19T-gRNA backbone plasmid as template. Followed purification using gel extraction kit the PCR products were used for gRNA synthesis using the TranscriptAid T7 High Yield Transcription Kit following the manufacturer’s constructions (K0441, Thermo Fisher Scientific, Waltham, MA, USA). Cas9 mRNA was synthesized from the pXT7-Cas9 plasmid using the T7 mMESSAGE mMACHINE mRNA Transcription Synthesis Kit (AM1344, Thermo Fisher Scientific) and purified using RNeasy Mini Kit (#74106, Qiagen, Hilden, Germany). The Cas9 mRNA and gRNA for vdra or vdrb were combined (500ng/ul Cas9 mRNA, 50ng/ul gRNA)for microinjection immediately before use (16). For genetyping, the PCR products amplified from the genomic DNA of fish were run in PAGE gel after denaturation and annealing. The PAGE gel was stained with ethidium bromide for 15 min before UVB imaging. The homoduplexes and heteroduplexes were separated with heteroduplexes being slower in mobility (17). The primers used in this study were listed in **Table 1**.

**Table 1:** The primers used in the study.

FOR GENOTYPING	NCBI accession No.	Forward primer(5′ - 3′)	Reverse primer(5′ - 3′)
***vdra***	NC_007134.7	CGGTGGACACCAAGCTGAACT	CTCCAGTAATTCGCTCCTCAGC
***vdrb***	NC_007117.7	GTGAGGGCTGCAAAGGCTTCTTCA	CCCACTGAGACTCCAGGTTAACTCA
FOR qPCR ANALYSIS			
***pgc1a***	XM_017357139.2	GATTGAGGAGAGCACAGT	TCACAAGTGTAGCGGTAG
***trpv6***	NM_001001849.2	GTCCCAAACTACAAAGGC	TGGTGGCAATAATCTCAA
***g6pca.1***	NM_001003512.2	TCCAAGTGGAGCATGACT	ATCCTGGTATTTGATCTGTAAG
***g6pca.2***	NM_001163806.1	TAGCAAAGGGAAGAAATCA	CTTTAGACTCGCACTGTAGAT
***pck1***	NM_214751.1	TGGAGGAGGAGTCAGTCAGC	CCACCACATACGGCGAGT
***chrebp***	KF713494.1	ACCCCGACATGACCTTCAAC	TGTGGCATCTCTGTGTTGCT
***insra***	NM_001142672.1	GCGTGGCAATAATCTGTTCT	CGTTGATAGTGGTGAGGGGG
***insulin***	NM_131056.1	GGTCCTGTTGGTCGTGTC	GTTGTAGAAGAAGCCTGTTGG
***gck***	NM_001045385.2	TGATATTGTGCGTCTGGCGT	GCTGCCTTCTTCTGACTGGA
***glut2***	NM_001042721.1	ATGTCTTCATCGTTTTTGCC	CAGCCTCTTCTTGAGGTTTG
***acsl1b***	NM_001003569.2	CTGACGAGTTCGGTCGAGTT	TGGCGTACCAGTATGCAGTG
***cpt1b***	NM_001328192.1	TGAGACGGATTCTTTCCGCT	TTCGCTAGGCTTGTTACTTGC
***cpt2***	NM_001007447.1	AATGGATTGGGTGCAACGTG	TGAGTTCTAACCTTCAGGCTCT
***lpl***	NM_131127.1	GGACGGTCACGGGTATGTTT	CGATTCCTGCAACATGAGCG
***fads2***	NM_131645.2	CCAATCAGAGCGAGCCTTCA	ACGCATTCAAAGTGCCACAA
***acc***	NM_001271308.1	ATGGCAGAGCAAGACTCCAC	CCTCTGCAGGTCGATACGTC
***elovl5***	NM_200453.2	TTTCGGCTAGAAGGAAAGCAGT	GAACCGAAAGTGGGAGGTG
***ppar-gama***	NM_131467.1	AGCGACAATGCTCCTTTT	CACTTCGATGACCCCGTA
***scd***	NM_198815.2	CCGACCCTCACAACTCAAACC	GACAACGAAGCACATCAACACC
***cyp2r1***	NM_001386362.1	GACACCTTTGCCCATTAT	CAGAAGTCCTCCCATTTT
***cyp24a1***	NM_001089458.1	AAAGAGGGCAGCTATCCTGA	CATGAGCTTCTGCTGGAAAG
***cyp27b1***	NM_001311791.1	AAGGCCGTCGTCAAGGAAAT	CTCGAGACGTGGCGTAATGA
***β-actin***	NM_131031.1	ACTCAGGATGCGGAAACTGG	AGGGCAAAGTGGTAAACGCT
***ef1α***	NM_131263	TACCCTCCTCTTGGTCGCTTT	ACCTTTGGAACGGTGTGATTG

### Micro CT imaging and relative bone density measurement

A 100-day-old male zebrafish was treated with an anesthetic (MS-222) and wiped off the water. The zebrafish is then attached to the foam using medical tape and placed in a scanning room. Whole zebrafish were scanned using an Micro CT system (SkyScan 1276, Bruker, Germany). The camera type is XIMEA MH110XC-KK-TP and the pixel size is 17.420um. The voltage and current are 55kV and 200uA respectively. Each constant motion scan resulted in 4668 projections over 180° with an exposure time of 175 ms per projection. The exposure is 517 ms. Each zebrafish is imaged after two or three scans, covering the head, belly and tail. The three-dimensional (3D) images of the bones were obtained by Skyscan CTAn software (v.1.1.7, Skyscan CTAn, Kontich, Belgium). At least three adults in each genotype were scanned.

The 100-day-old zebrafish was anesthetized, the tissue was removed, and the spine, about 20-25 sections, was obtained. The spine were scanned using an Micro CT system (SkyScan 1276, Bruker, Germany). Each constant motion scan resulted in 686 projections. The parameters are consistent with Micro CT. Each spine is imaged after two scans. The Skyscan CTAn software was used to calculate the bone density of each section and the bone density of all sections of the spine was averaged. At least three adults in each genotype were scanned.

### Total RNA extraction and quantitative real-time PCR

The gill, adipose tissue and liver were dissected from zebrafish under the anaesthetic. Total RNA was extracted with 100mg gill, 60mg adipose tissue and 100mg liver tissue dissected 100-day-old male zebrafish in 1ml TRIzol reagent (Ambion, Austin, TX). Generally, RNA with a content of 1-3ug is used for cDNA synthesis. The cDNAs were synthesized using the Reverse Aid First-Strand cDNA Synthesis Kit (K1622, Thermo Scientific, Waltman, MA, USA) according to the manufacturer’s instructions. Quantitative real-time PCR primers were designed using “primer blast” in the National Center for Biotechnology Information (NCBI) database, and are listed in **Table 2**. The primers for qPCR were validated by DNA sequencing and agarose gel electrophoresis of PCR products. RT-qPCR was conducted using TransStart^®^ Tip Green qPCR SuperMix (Transgen, China) with the Bio-Rad (CFX96 Touch) software. All mRNA levels were calculated as fold expression relative to the housekeeping gene, β-actin. Each sample was run in triplicate repeats and the results were calculated according to the method described previously (18). In order to avoid post-feeding interference, no feeding was performed from 4 pm the previous day until 8 am next day. All of the following genes were used the tissues of zebrafish in the fasting state except the chrebp and gck.

**Table 2:** The levels of 1α,25(OH)_2_VD_3_ and free fatty acid.

TEST	Sample or treatment	Con	vdra-/-;vdrb-/-	vdra-/-	vdrb-/-
**1,25(OH)2VD3**	**Plasma(pg/ml)**	**161.0 ± 8.936a**	**480.3 ± 43.00c**	**259.6 ± 30.33b**	**345.9 ± 42.02b**
	**Liver(pg/g)**	**218.6 ± 2.005a**	**1633.0 ± 69.69c**	**352.8 ± 21.12b**	**409.8 ± 46.77b**
	**Adipose(pg/g)**	**657.8 ± 10.64a**	**69.4 ± 4.342c**	**456.9 ± 12.79b**	**130.2 ± 6.11c**
**Free Fatty Acid**	**Plasma(μmol/L)**	**721.5 ± 50.9a**	**780.1 ± 55.65a**	**845.3 ± 69.85a**	**777.1 ± 66.85a**

* n=3 groups, 15-20 fish/group.

### Mineral content measurement

Plasma of 100-day-old male zebrafish were used for nitration with nitric acid (HNO3). The lysate was then diluted to 10 mL with 2% nitric acid solution. The calcium and phosphorus levels were measured using inductively coupled plasma-optical emission spectroscopy (ICP-OES) on a Perkin-Elmer Optimal 8000 (Perkin Elmer, MA, USA). ICP-OES calibration standards were prepared from stock solutions on a gravimetric basis. Three target calibration standards were used for each ion, and 2% HNO3 in double-deionized water was used as a control.

### Whole-mount *in situ* hybridization

The experiment was carried out according to Chou’s method (19). Zebrafish gills were fixed with 4% paraformaldehyde overnight at 4°C, and then washed several times with phosphate-buffered saline (PBS). Fixed samples were rinsed with PBST (PBS with 0.2% Tween 20, 1.4 mmol l – 1 NaCl, 0.2 mmol l – 1 KCl, 0.1 mmol l – 1 Na2HPO4, and 0.002 mmol l – 1 KH2PO4; pH 7.4). After a brief washing with PBST, gill filaments were incubated with hybridization buffer (HyB) containing 60% formamide, 5x SSC, and 0.1% Tween 20 for 5 min at 65°C. Prehybridization was performed in HyB+ (the hybridization buffer supplemented with 500 μg ml – 1 yeast tRNA and 50 μg ml – 1 heparin) for 2 h at 65°C. After prehybridization, samples were hybridized in 100 ng of the RNA probe in 200 μl of HyB+ at 65°C overnight. Gills were then washed at 65°C for 10 min in 75% HyB and 25% 2x SSC, for 10 min in 50% HyB and 50% 2x SSC, for 10 min in 25% HyB and 75% 2x SSC, for 10 min in 2x SSC, and twice for 30 min each in 0.2x SSC at 70°C. Further washes were performed at room temperature for 5 min in 75% 0.2x SSC and 25% PBST, for 5 min in 50% 0.2x SSC and 50% PBST, for 5 min in 25% 0.2x SSC and 75% PBST, and for 5 min in PBST. After serial washings, gill filaments were incubated in blocking solution containing 5% sheep serum and 2 mg ml – 1 BSA in PBST for 2 h and then incubated in the 1:10 000 - diluted alkaline phosphatase-conjugated anti-dig antibody for another 16 h at 4°C. After the reaction, samples were washed with PBST plus blocking reagent and then stained with NBT and BCIP.

### Nile Red staining

Nile Red (N3013; Sigma Aldrich, St. Louis, MO, USA) was dissolved in acetone at 1 mg/mL as the stock solution. Mutants and size-matched control zebrafish were immersed in system water containing 0.1 μg/mL Nile Red for 1 hr for larval fish. Images were obtained using an Olympus SZX16 FL Stereo Microscope (Olympus, Tokyo, Japan) at an excitation wavelength of 488 nm.

### Body fat ratio assay

Twelve adult zebrafish were selected randomly from the three mutant lines and controls. All samples were frozen in liquid nitrogen and put into the -80°C refrigerator overnight. Subsequently, the samples were lyophilized in vacuum drying equipment at -20°C for 24 h. Bodyweight was recorded before fat extraction in methanol and chloroform. The oil extracted from each fish was weighed. The total oil weight/dry body weight ratios were calculated.

### Measurement of triglyceride, glycogen, lactic acid and free fatty acid

The triglyceride, glycogen, lactic acid levels in the livers of 100-day-old male zebrafish were measured using the Triglyceride Assay Kit (Item No. A110-1-1), Liver/Muscle Glycogen Assay Kit (Item No. A043-1-1), Lactic Acid Assay Kit (Item No. A019-2-1) purchased from Nanjing Jiancheng Bioengineering Institute. The tail of the zebrafish was cut off after anesthesia, and blood was collected from the severed tail with a micropipette gun rinsed with heparin sodium. Then the zebrafish was dissected to collect liver tissue. Each parallel sample required about 15mg of liver tissue or 10ul of plasma for the corresponding indicator detection. At least 3 parallel samples are required for each indicator. The detection kit for lactic acid in plasma is consistent with the detection kit for lactic acid in liver. The plasma FFA levels were measured using the Nonesterified Free fatty acids assay kit (Item No. A042-2-1) purchased from Nanjing Jiancheng Bioengineering Institute. The samples were processed following the procedure provided by the manufacturer. Each parallel sample required 4 ul of plasma, and the experiment was repeated eight times.

### Measurement of 1α,25(OH)2VD3 levels

Plasma was prepared from blood for measurement of 1α,25(OH)_2_VD_3_ levels. 1α,25(OH)_2_VD_3_ (Item No. D1530-10UG) and 4-phenyl-1,2,4-triazolin-3,5-dione (Item No. 42579-100MG) were purchased from Sigma Aldrich and used as standards. 1α,25(OH)_2_VD_3_ was extracted from plasma and measured as according to a previous report (8) using ultra-performance liquid chromatography-triple quadrupole mass spectrometry (UPLC-TQMS) (ACQUITY UPLC System; Waters Corp, Milford, MA, USA). Quantification was performed using calibration curves generated with the 1α,25(OH)_2_VD_3_ standard. Calibrations and sample measurements were performed in triplicate.

### Blood glucose measurement

The OneTouch UltraVue (LifeScan, Inc., Milpitas, CA, USA) glucometer was used for the measurement of blood glucose. Zebrafish were fed regularly with a chow diet and then fasted for 18 h prior or were fed normally for 2 h prior to removal of blood using a heparin-rinsed micropipette tip, as described previously (20). Student’s *T*-test was used to evaluate blood glucose levels at different time points.

### Western blotting

Protein samples were collected from the liver tissue of zebrafish at 100 dpf. The total protein of the liver tissue was extracted from 100mg liver tissue in 500 micro liter lysis buffer for each sample. For western blot analyses, sodium dodecyl sulfate-polyacrylamide gel electrophoresis (SDS–PAGE) was prepared as previously described (21). Total protein was separated by SDS–PAGE and transferred onto polyvinylidene difluoride membranes (Bio-Rad). Primary antibodies including anti-β-actin (AC026; ABclonal Technology, Woburn, MA, USA), anti-Akt (9272S; Cell Signaling Technology, Inc., Danvers, MA, USA), anti-phospho-Akt (Ser473) (4060S; Cell Signaling Technology, Inc.), anti-S6 ribosomal protein 5G10 (2217S; Cell Signaling Technology, Inc.) and anti-phospho-S6 (Ser235/236) (4858S; Cell Signaling Technology, Inc.) were diluted at 1:1,000 in Can Get Signal antibody dilution solution (NKB-101; Toyobo Co., Ltd., Osaka, Japan). Signal was detected using a charge-coupled device camera-based imager (ImageQuant LAS 4000 mini; GE Healthcare, Chicago, IL, USA). The intensity of the bands was quantified using ImageJ version 1.49V (22) with β-actin, S6, or AKT serving as controls.

## Results

### Generation of zebrafish *vdra* and *vdrb* deficient using CRISPR/Cas9 technique

**Figure 1:**
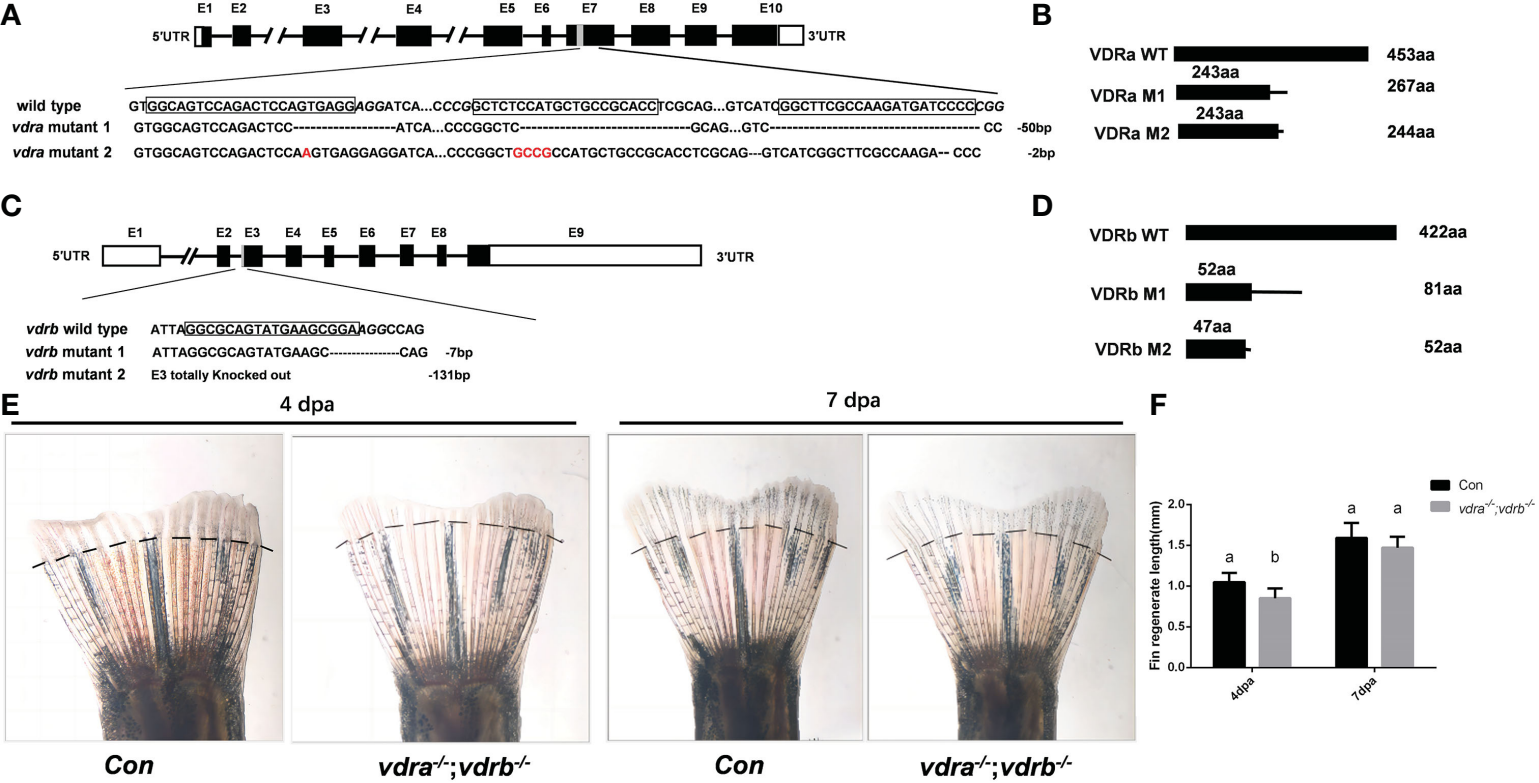
Generation of zebrafish *vdra*- and *vdrb* –deficient using CRISPR/Cas9 technique. **(A)** Targeted depletion of the *vdra* gene; The CRISPR/Cas9 target site was located in exon 7. Two genotypes, 50-bp deletion and 2-bp deletion, were used to establish the *vdra* knockout line (highlighted in red); **(B)** The diagram shows the predicted VDRa protein from wild-type, M1, and M2 zebrafish. M1 consisted of 243 amino acids identical to WT (green) and 24 miscoding amino acids (black); M2 consisted of 243 amino acids identical to WT and one miscoding amino acid (black); **(C)** Targeted depletion of the *vdrb* gene; The CRISPR/Cas9 target site was located in exon 3. Two genotypes, 7-bp deletion and 131-bp deletion, were used to establish the *vdrb* knockout line (highlighted in red); **(D)**. The diagram shows the predicted VDRb protein from wild-type, M1, and M2 zebrafish. M1 consisted of 52 amino acids identical to WT (green) and 29 miscoding amino acids (black); M2 consisted of only 47 amino acids identical to WT and five miscoding amino acids (black). **(E)** Zebrafish(100dpf) caudal fin regeneration at 4 and 7days post amputation. Dashed lines indicated approximate amputation planes. **(F)**. Statistical analysis of the regeneration length. Six fish were used in each group. The bars with different letters indicated significant difference(p<0.05).

To investigate the function and regulation mechanisms of VD_3_/VDR signaling, *vdra* and *vdrb* were knocked out using CRISPR/Cas9. The target of CRISPR/Cas9 for *vdra* was located in exon 7, and target of the CRISPR/Cas9 for *vdrb* was located in exon 3. F_0_ embryos were injected with Cas9-mRNA and guide RNA (gRNA). Genomic DNA were extracted from some of the injected F_0_ embryos for genotyping. The remaining F_0_ embryos were allowed to develop and reproduce. The F_1_ generation was genotyped and deep sequenced. Then, two mutant lines for *vdra* and *vdrb* were established. The altered genetic sequences of *vdra* and *vdrb* are shown in **Figures 1A, C**. The mutant genomic sequences of *vdra* and *vdrb* alleles produced premature proteins (**Figures 1B, D**). Since there was low universality of the mammal VDR antibody to detect zebrafish VDRs protein. The fin regeneration was examined and the phenotype was verified accordance with previous investigation (**Figures 1E, F**). Vitamin D receptors ablation inhibited regeneration of amputated fins as previously described.

### Nuclear receptors of VD_3_ is not essential for calcium homeostasis in zebrafish

**Figure 2:**
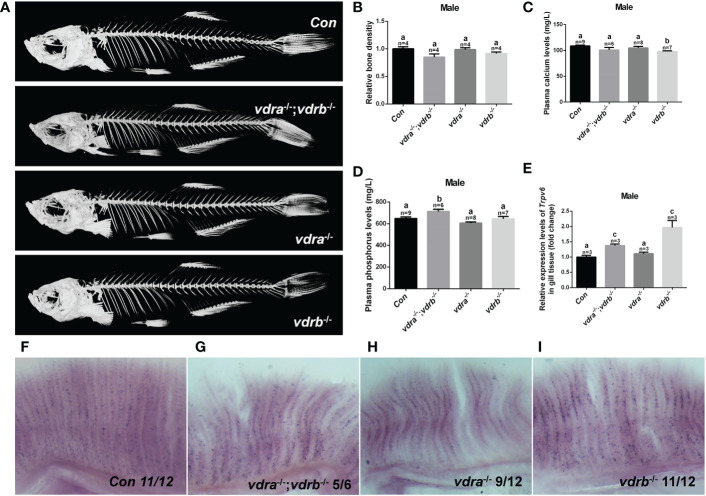
The activities of *vdra* and *vdrb* are dispensable for the calcium homeostasis and bone development. **(A)** Micro CT scanning images of mutants and control siblings at 100 dpf; **(B)** the relative bone density examined using CT scanning. **(C)** Calcium and **(D)** phosphorus levels in plasma were measured in mutants and control siblings at 100 dpf; **(E)** Quantitative real-time PCR assays show the *trpv6* transcript levels in the gills of mutants and control siblings at 100 dpf. **(F–I)**. The trpv6 expression patterns in the gill of the mutant and control. The representative images were selected and the blue-purple dot in the gills was the expression signals. The bars with different letters indicated significant difference (p<0.05).

Vitamin D_3_, in particular its active metabolite 1α,25(OH)_2_VD_3_, is widely known as a modulator of calcium and phosphate homeostasis. Deficiency in VD_3_ or VDR signaling will cause impaired bone mineralization. To further determine whether VDRs are necessary for calcium, phosphate homeostasis and mineralization of the bone, micro-computed tomography (CT) imaging was performed. The images showed that there were no significant defects in the mineralization of the vertebral column, ribs, craniofacial bone and fins in VDR mutants (**Figure 2A**). The relative skeletal bone density was examined and caculated. There were no statistic significant difference between *vdra* ^-/-^;*vdrb* ^-/-^, *vdra^-/-^*, *vdrb* ^-/-^ and control zebrafish although the bone density of *vdra* ^-/-^;*vdrb* ^-/-^ was moderately decreased (**Figure 2B**). Calcium and phosphate levels were also not significantly changed compared with controls (**Figures 2C, D**). The calcium transporter protein TRPV6, of which the promoter contains a VDR element, served as a 1α,25(OH)_2_VD_3_/VDR target gene. Expression levels of *trpv6* were checked using real-time PCR. Corresponding with calcium levels, the expression of *trpv6* was retained in the gills. The expression of *trpv6* was even enhanced in *vdra* ^-/-^;*vdrb* ^-/-^ and *vdrb* ^-/-^ fish (**Figure 2E**). For better view of *trpv6* expression pattern in gills, *in situ* hybridazation was performed. According with the relative quantification data, the trpv6 expression was increased in the vdrb ablation and *vdra* ^-/-^;*vdrb* ^-/-^ double knockout fish (**Figures 2F–I**).

### Growth retardation and accumulated adiposity in the *vdra^-/-^*; *vdrb^-/-^* zebrafish

**Figure 3:**
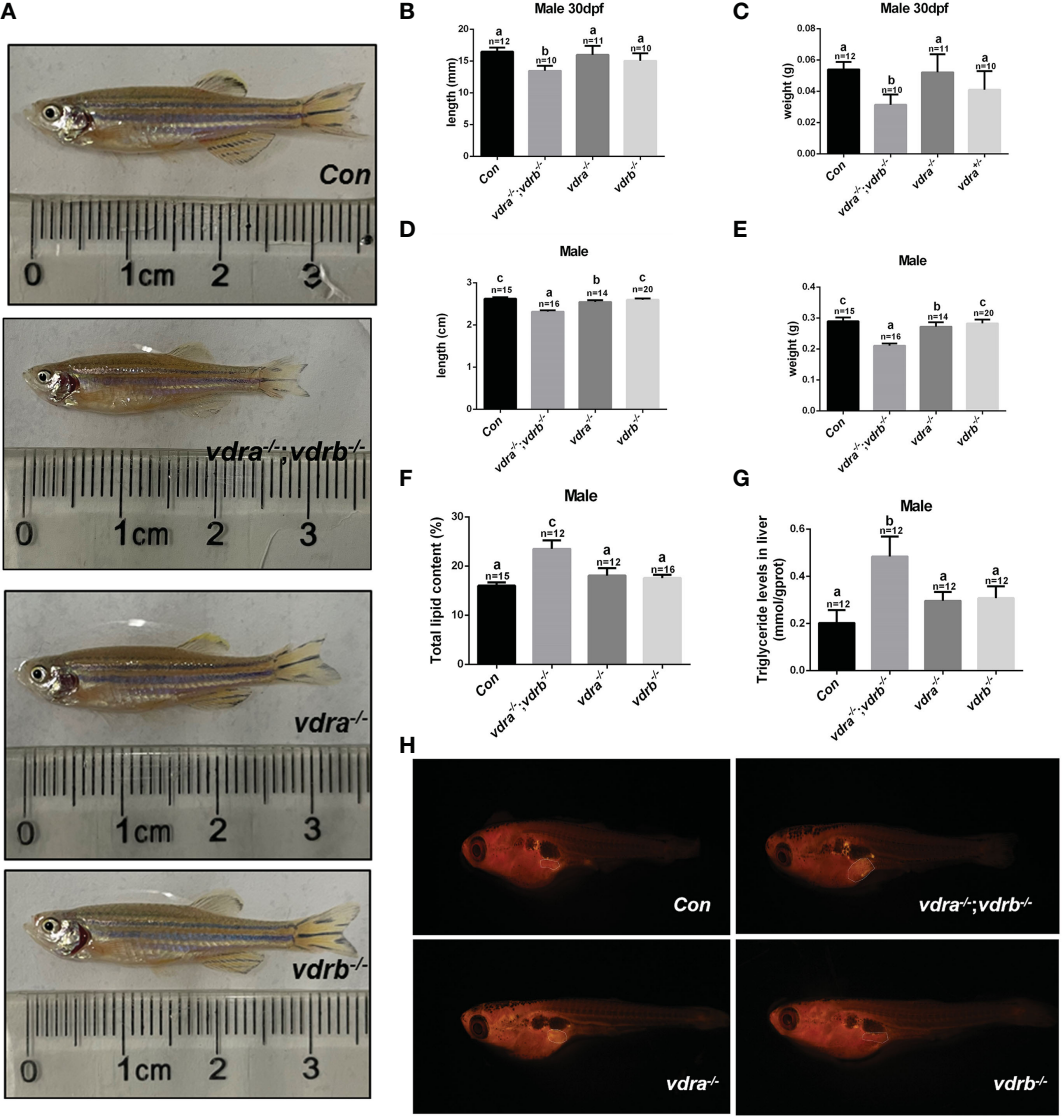
Retarded growth and accumulated adiposity observed in *vdra^-/-^*;*vdrb^-/-^* zebrafish. **(A)** Comparison of the morphological features of mutants and control siblings at 100 dpf; **(B–E)** The body length and weight of the mutant lines and control siblings. **(B, C)** showed the zebrafish at 30dpf and **(D, E)** showed the zebrafish at 100dpf. **(F)** The total lipid levels of male mutants and controls at 100 dpf; controls, n = 15; *vdra^-/-^;vdrb^-/-^*, n = 12; *vdra* ^-/-^;*vdrb* ^+/-^, n = 12; *vdra* ^+/-^;*vdrb* ^-/-^, n = 16; **(G)** The levels of triglycerides in the liver of mutants and control siblings at 100 dpf; **(H)**. Neutral lipids of juvenile mutants and size-matched controls (both with a standard length of 7.6 mm) were stained with Nile Red and viewed with stereoscope. The visceral adipose tissue was labeled by the surrounded dashed line. The bars with different letters indicated significant difference (p<0.05).

Our previous study demonstrated that 1α,25(OH)_2_VD_3_ was required for lipid metabolism and adipose tissue homeostasis (8). 1α,25(OH)_2_VD_3_ deficiency caused by knockout of *cyp2r1* gave rise to drastic growth retardation. In the present study, *vdra*-deficient zebrafish showed slightly reduced growth, while *vdrb*-deficient zebrafish showed normal growth compared with controls (**Figures 3A–E**). The double knockout of *vdra* and *vdrb* exhibited more severe growth retardation because of elimination of functional redundancy (**Figures 3A–E**). Besides the features of lipid metabolism in *vdr*-deficient fish were analyzed. The whole mount triglyceride levels of adult *vdra* ^-/-^;*vdrb* ^-/-^ zebrafish was significantly higher compared with control zebrafish (**Figure 3F**). As a main location of lipid metabolism, triglyceride levels in the liver were measured. Consistent with the TG content in the entire body, *vdra* ^-/-^;*vdrb* ^-/-^ fish exhibited higher triglycerides in the liver compared with the controls (**Figure 3G**). The Nile-Red-stained visceral fat of larvae fish was viewed using stereoscope with size-matched

mutant fish and control fish. The visceral adipose tissue significantly increased in *vdra* ^-/-^;*vdrb* ^-/-^ line compared with control fish (**Figure 3H**). Therefore, the *vdra* ^-/-^;*vdrb* ^-/-^ line, in which the 1α,25(OH)_2_VD_3_ nuclear receptor was substantially deficient, served as the main model used in subsequent investigations.

### VDRs ablation impair lipid and glucose homeostasis in liver and adipose tissue

**Figure 4:**
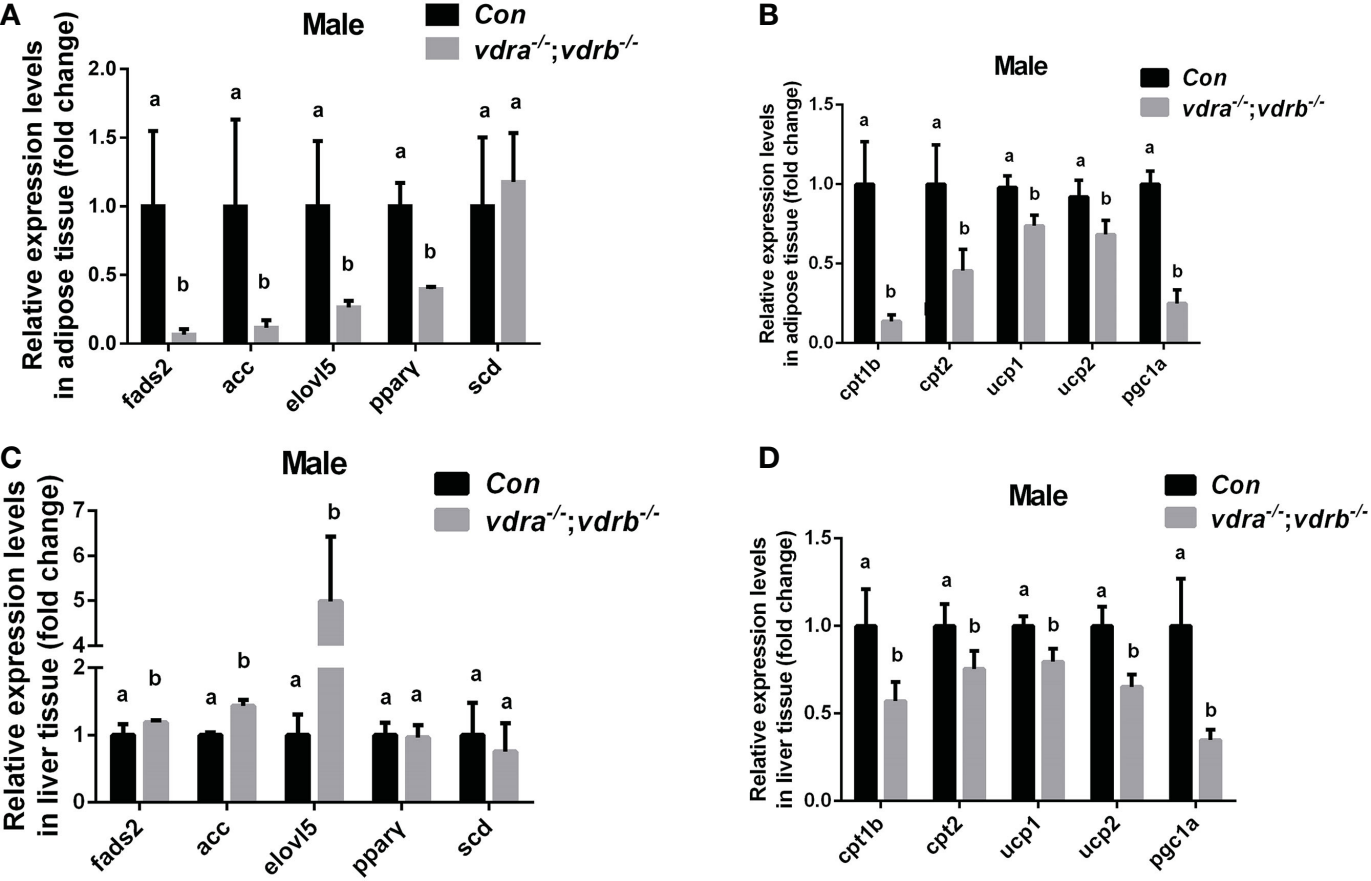
Transcriptional levels of maker genes involved in the lipogenesis and lipid oxidation. **(A, C)** Relative expression levels of marker genes involved in lipogenesis including *fads2,acc,elovl5,pparγ* and *scd* in adipose tissue **(A)** and liver tissue **(C)** of mutant line and control. Three biological repeats were carried out and statistical analysis was performed using a t test (n=3). **(B, D)** Relative expression levels of *cpt1b, cpt2,ucp1, ucp2* and *pgc1α* in adipose tissue **(A)** and liver tissue **(C)** of mutant line and control. Three biological repeats were carried out and statistical analysis was performed using a t test (n=3). There was no difference between the two internal reference gene (*β-actin* and *EF1α*). The bars with different letters indicated significant difference (p<0.05).

Since the observation of the fatty liver and increased visceral adiposity, we checked the lipid metabolism state in liver and adipose tissue. The free fatty acid (FFA) was measured and there was no significant difference between *vdra* ^-/-^;*vdrb* ^-/-^ line and control line (**Table 2**). *Δ-6-fatty acid desaturase* (*fads2*) is the key enzyme in the biosynthesis of polyunsaturated fatty acids (PUFAs). *Acetyl-CoA carboxylase*(*ACC*) is a biotin-dependent enzyme that catalyses the irreversible carboxylation of acetyl-CoA to malonyl-CoA, the rate-limiting step in fatty acid biosynthetic pathway. *Elongases of very long-chain fatty acids 5*(*elovl5*) are the initial and rate-limiting enzymes responsible for the condensation of activated fatty acids with malonyl-CoA required for biosynthesis of long-chain fatty acids. *Peroxisome proliferator-activated receptor gamma* (*pparγ*) as the lipid synthesis regulator,enhanced lipogenesis through upregulation of at both transcript and protein levels. *Stearoyl-CoA desaturase* (*SCD*) is known for their roles in synthesizing unsaturated fatty acids. The lipogenesis pathway was enhanced in the liver with the elevated transcription levels of *fads2, ACC* and *elovl5*. While in the adipose tissue the lipogenesis pathway was suppressed with the decreased transcription levels of f*ads2,ACC,elovl5* and *pparγ* (**Figures 4A, C**). Carnitine palmitoyltransferase (CPT) catalyze the conversion of fatty acylCoAs into fatty acylcarnitines for entry into the mitochondrial matrix, it is deemed as the main regulatory enzyme in mitochondrial fatty acid β-oxidation. The expression of *pgc1α*, which is a modulator of beta-oxidation, was significantly suppressed (**Figures 4B, D**). The lipolysis pathway was down-regulated in the adipose and liver tissue with the decreased transcription levels of *cpt1b, cpt2*, and *pgc1α*. The mitochondria uncoupling proteins were belonged to super mitochondria carrier superfamily with a general role in protection against oxidative stress. And the *ucps* were stimulated by fatty acid oxidation. The transcript levels of some mitochondria-related genes, such as *ucp1* (uncoupling protein 1) and *ucp2*(uncoupling protein 2) were suppressed (**Figures 4B,D**). This suggested that the elevated triglyceride accumulation in adipose tissue was attributed to defects in triglyceride mobilization. While in the liver the beta-oxidation was suppressed and lipogenesis was promoted.

**Figure 5:**
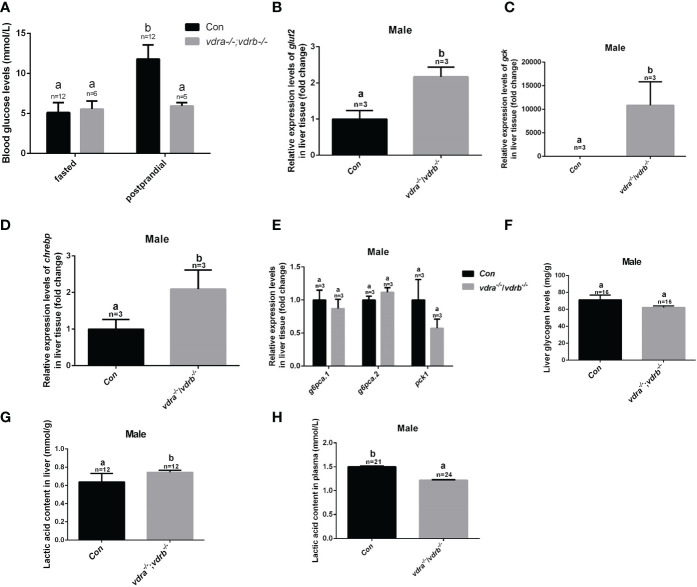
Glucose metabolic homeostasis was impaired in the liver of *vdra^-/-^*;*vdrb^-/-^* zebrafish. **(A)** Blood glucose levels of *vdra^-/-^;vdrb^-/-^* fish and control siblings in the fasted state and postprandial state at 100 dpf. **(B–D)**. Quantitative real-time PCR showed *glut2, gck and chrebp* transcriptional levels in the liver of mutants and control siblings at 100 dpf; **(E)** Quantitative real-time PCR showed gluconeogenesis marker gene *g6pca.1, g6pca.2* and *pck1* transcriptional levels in the liver of the *vdra* ^-/-^;*vdrb^-/-^* fish and control siblings at 100dpf. **(F)** The liver glycogen levels in *vdra* ^-/-^;*vdrb* ^-/-^ fish and control siblings at 100 dpf. **(G, H)**. The liver lactic acid levels in *vdra* ^-/-^;*vdrb* ^-/-^ fish and control siblings at 100 dpf. The bars with different letters indicated significant difference (p<0.05).

In the previous investigation, 1α,25(OH)_2_VD_3_ signaling regulates insulin signaling through modulating the expression of the insulin receptor. 1α,25(OH)_2_VD_3_-deficient zebrafish exhibited impaired blood glucose homeostasis and hyperglycemia (11). Therefore the blood glucose levels of VDRs ablation zebrafish were checked. The fasting blood glucose levels were indistinguishable between the control zebrafish and the *vdra* ^-/-^;*vdrb* ^-/-^ zebrafish. However the postprandial blood glucose levels of the *vdra* ^-/-^;*vdrb* ^-/-^ line was decreased compared with control line (**Figure 5A**). The data suggested that the glucose tolerance was ameliorated in the *vdra* ^-/-^;*vdrb* ^-/-^ zebrafish. This speculation was supported by the increased expression of *glut2* and *gck*, which are markers for glycolysis and glucose transportation into cells (**Figures 5B, C**). The expression of *chrebp*, which encodes a key positive regulator of lipid synthesis, was increased (**Figure 5D**). This suggested that lipid synthesis from glucose was promoted in the liver. Although there was increased glycolysis, gluconeogenesis was unaffected (**Figure 5E**). The expression levels of key regulators and enzymes of gluconeogenesis such as *pck1*, *g6pca.2*, and *g6pca.1* in the liver were unaffected in *vdra^-/-^;vdrb^-/^* ^-^ fish (**Figure 5E**). To determine effects on the metabolic pathways of glucose, metabolites such as glycogen and lactic acid were measured. The synthesis of lactic acid and glycogen were elevated in the liver, which revealed increased liver glucose catabolism (**Figures 5F–H**).

### Elevated 1α,25(OH)2VD3 levels and Enhanced insulin signaling activity in the *vdra^-/-^*; *vdrb^-/-^* zebrafish

**Figure 6:**
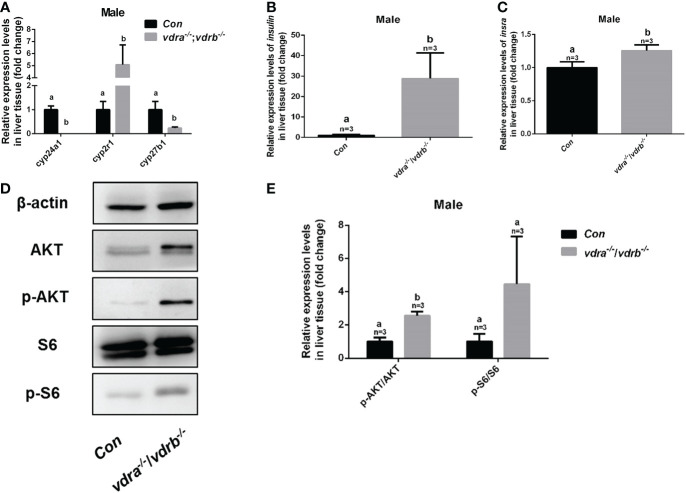
Enhanced insulin signaling observed in *vdra^-/-^*;*vdrb^-/-^* zebrafish. **(A)** Quantitative real-time PCR showed the *cyp24a1, cyp2r1* and *cyp27b1* transcript levels in the liver of *vdra^-/^;vdrb^-/-^* fish and control siblings at 100 dpf; **(B, C)** Quantitative real-time PCR showed the *insulin and insra*, transcriptional levels in the liver of *vdra^-/^;vdrb^-/-^* fish and control siblings at 100 dpf; **(D)** AKT, p-AKT, S6, and p-S6 protein levels in liver samples of *vdra^-/-^;vdrb^-/-^* fish and control siblings at 100 dpf; **(E)** Quantification of relative p-AKT/AKT and p-S6/S6 protein levels from western blot analysis.

To investigate the mechanisms of VDR involvement in insulin signaling, 1α,25(OH)_2_VD_3_ levels were monitored using ultra high-performance liquid chromatography-triple quadrupole mass spectrometry (UPLC-TQMS). The levels of 1α,25(OH)_2_VD_3_ were significantly elevated compared with controls in the plasma and liver (**Table 2**). However the levels of 1α,25(OH)_2_VD_3_ in the adipose tissue was significantly suppressed. It has been suggested that ablation of the *VDRs* would promote ligand production as a feedback effect. The major enzyme encoding genes responsible for 1α,25(OH)_2_VD_3_ homeostasis including cyp2r1, cyp27b1 and cyp24a1 were tested. The expression of cyp24a1 was significantly reduced. The expression of cyp27b1was suppressed. However the expression of cyp2r1which was responded for 25(OH)VD3 synthesis was substantially promoted (**Figure 6A**). Intriguingly, the expression levels of insulin and insulin receptor were significantly increased in the liver of *vdra^-/-^;vdrb^-/-^* zebrafish(**Figures 6B, C**). Due to the increased insulin signaling and 1α,25(OH)_2_VD_3_ levels, the activity of the AKT/mTOR pathway was also measured, showing increased levels of phosphorylated AKT and S6 proteins (**Figures 6D, E**). In summary, *vdra^-/-^;vdrb^-/-^* zebrafish showed elevated 1α,25(OH)_2_VD_3_ levels in the liver, and this promoted insulin/insulin-receptor signaling. Therefore, the augmentation of insulin activity enhanced glucose absorption and lipid synthesis.

## Discussion

Insights into the physiological actions of 1α,25(OH)_2_VD_3_ in zebrafish have been gradually achieved from recent studies in several deficient zebrafish models (5–11). Basically, the involvement of 1α,25(OH)_2_VD_3_ in mineral ion homeostasis or related craniofacial cartilage development in zebrafish have been only observed in several studies using morpholino-based knockdowns (5, 10). However, no effects on physiological calcium handling have been seen in several 1α,25(OH)_2_VD_3_ signaling-deficient zebrafish models (8, 9), which is similar to the phenotypes seen in the present study. This suggests that calcium homeostasis might not necessarily be dependent on the nuclear VDR in teleosts.

Surprisingly, no significantly defective calcium homeostasis or skeletal development was seen in *cyp2r1*-deficient (8) and *vdra* ^-/-^;*vdrb* ^-/-^ zebrafish (**Figure 2**). Moreover, no such defects has been stated in other *vdra* ^-/-^;*vdrb* ^-/-^ zebrafish model either (9). The trpv6 was reported to be the downstream effector of vitamin D3/VDR signaling. The expression of trpv6 in the gill was upregulated in *vdra* ^-/-^;*vdrb* ^-/-^ and *vdrb^-/-^* zebrafish. It indicated that the trpv6 expression induction was VDRs independent. In the 1α,25(OH)_2_VD_3_ deficient model the trpv6 expression was not affected both larval and adult stage. the vitamin D3/VDR signaling was not essential for the Ca^2+^ absorption in teleost fish. Both VDRa and VDRb were functional redundant. Double knockout of the VDRs caused more severe growth retardation and adiposity. However, similar phenotypes regarding retarded growth, increased obesity, and decreased *pgc1α* expression have been seen in both *cyp2r1* ^-/-^ and *vdra* ^-/-^;*vdrb* ^-/-^ zebrafish (8). The involvement of 1α,25(OH)_2_VD_3_ signaling in fatty acid metabolism has also been reported (4, 23). The levels of 1α,25(OH)_2_VD_3_ were significantly elevated in *vdra^-/-^;vdrb^-/-^* zebrafish due to a feedback mechanism. The mitochondria beta oxidation related genes were suppressed significantly in the *vdra* ^-/-^;*vdrb* ^-/-^ zebrafish. Therefore, the decreased lipid oxidation can be attributed to the impaired downstream VDR signaling.

In zebrafish, two types of nuclear VDRs have been identified (12). Compared with the differences between the *cyp2r1* ^-/-^ zebrafish and *vdra* ^-/-^;*vdrb* ^-/-^ zebrafish in terms of the body weight and length, milder defects were seen in *vdra^-/-^;vdrb^-/-^* fish compared to control siblings at 100 days post-fertilization (8). Considering that about half of the plasma content of 1α,25(OH)_2_VD_3_ still remained in *cyp2r1* ^-/-^ zebrafish (8), it is reasonable to speculate that vitamin D_3_ signaling can act in a nuclear VDR-independent manner. Intriguingly, decreased insulin levels were seen in *cyp2r1* ^-/-^ zebrafish with reduced 1α,25(OH)_2_VD_3_ levels (11). However, significant upregulation of insulin transcriptional and AKT activation have been seen in the liver of the present *vdra* ^-/-^;*vdrb* ^-/-^ zebrafish. A previous study, in which diet-supplemented vitamin D_3_ could promote insulin signaling supports this speculation (11). Moreover, significant enhancement of glucose catabolism was also seen in our *vdra* ^-/-^;*vdrb* ^-/-^ zebrafish. Augmentation of insulin signaling promoted glucose uptake, glycolysis and lipid synthesis revealed by the increased expression of *glut2, gck* and *chrebp.* In view of the elevated plasma levels of 1α,25(OH)_2_VD_3_ presented in *vdra* ^-/-^;*vdrb* ^-/-^ zebrafish, it could be promotion of the high levels of the 1α,25(OH)_2_VD_3_ on insulin signaling with the receptor(s) other than VDRa or VDRb in zebrafish.

Protein disulfide isomerase family A member 3 (pdia3), also known as 1α,25(OH)_2_VD_3_-membrane-associated rapid response to steroid, has been described as a crucial protein in 1α,25(OH)_2_VD_3_-initiated rapid membrane non-genomic signaling pathways in mammals (24, 25). No significant alterations in transcriptional expression levels of *pdia3* were observed in the hepatic tissue of our *vdra* ^-/-^;*vdrb* ^-/-^ fish (data not shown). However, the majority of the responses mediated by vitamin D_3_ non-genomic signaling have implicated post-translational modification (24, 26, 27). It has been reported that *pdia3* might have a significant role in calcium absorption, skeletal development, lipogenesis, and insulin regulation (24–27).

The lack of available data on the non-genomic actions of vitamin D_3_ stands in the way of understanding the mode of actions of vitamin D_3_ metabolites. Further studies will need to be performed to revealed the modes of action of vitamin D_3_ across its evolutionary history using teleost models.

## Conclusion

In the present study we utilized *vdra* ^-/-^;*vdrb* ^-/-^ double knockout zebrafish model to characterize the function of VD3/VDRs signaling. Our results demonstrated VDRs were nor essential for calcium homeostasis and skeleton development. The 1α,25(OH)_2_VD_3_ levels were elevated through cyp24a1 transcriptional repression. The lipolysis was not affected in the *vdra* ^-/-^;*vdrb* ^-/-^ zebrafish. However the lipid oxidation activities were inhibited due to the VDRs ablation. Furthermore enhanced transcription level and activity of Insulin/Insr were achieved in the *vdra* ^-/-^;*vdrb* ^-/-^ zebrafish. Augumentation of the glucose uptake, glycolysis and lipogenesis intensified the lipid accumulation in the liver.

## Data availability statement

The original contributions presented in the study are included in the article/**Supplementary Material**. Further inquiries can be directed to the corresponding author.

## Ethics statement

All animal experiments were conducted in accordance with the Guiding Principles for the Care and Use of Laboratory Animals and were approved by the Institute of Hydrobiology, Chinese Academy of Sciences (Approval ID: IHB 2013724).

## Author contributions

RL conducted most of the experiments for this study. XP and YL provided guidance in experimental operation. XJ, JH, and GZ helped in maintaining the fish system technical guidance. QL and ZY performed training and provided insights for this work. QL and ZY wrote the paper and prepared the figures. ZY initiated and supervised the research team and edited the paper. JJ, YR, SS, TS, and TL gave valuable suggestion and discussion. All authors contributed to the article and approved the submitted version.
