# Supplementary figures and images for: Enhanced insulin activity achieved in VDRa/b ablation zebrafish
Source: Front Endocrinol (Lausanne). 2023 Feb 13;14:1054665. doi: 10.3389/fendo.2023.1054665 (PMC9972578; doi:10.3389/fendo.2023.1054665)

## Slide 1
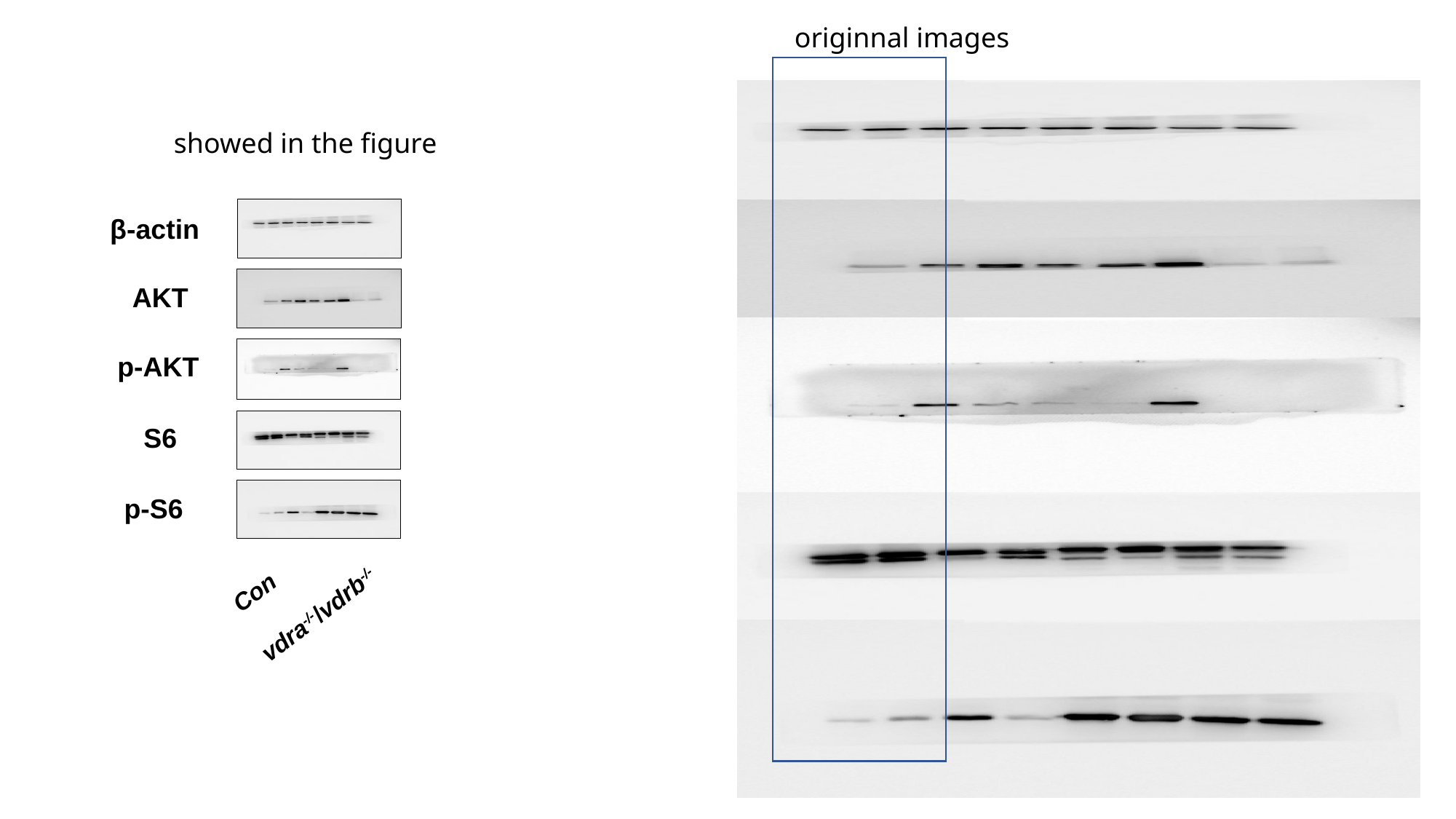

originnal images
showed in the figure
β-actin
AKT
p-AKT
S6
p-S6
Con
vdra-/-/vdrb-/-

Supplement: Supplementary file 1 [file Presentation_1.pptx]
